# Precision Phenotypic Profiling and Capture of Circulating Tumor Cells via a Vertical Laminar Flow-Stacked Microfluidic Chip

**DOI:** 10.3390/mi15040542

**Published:** 2024-04-18

**Authors:** Xinping Zhang, Yuan Ma, Yujiao Wang, Zhenwei Liang, Xuanhe Zhang, Yiqing Chen, Qingyi Wang, Hua Qin, Jiadao Wang

**Affiliations:** 1 School of Mechanical-Electronic and Vehicle Engineering, Beijing University of Civil Engineering and Architecture, Beijing 102616, China; 2 Department of Mechanical Engineering, Tsinghua University, Beijing 100084, China

**Keywords:** circulating tumor cells, microfluidic chip, magnetic cell sorting, subtype sorting

## Abstract

The heterogeneity of circulating tumor cells has a significant impact on the diagnosis, treatment, and monitoring of cancer. Research on the subtypes of circulating tumor cells can bring better treatment outcomes for cancer patients. Here, we proposed a microfluidic chip for the magnetic capture of subtypes of circulating tumor cells from the whole blood and phenotypic profiling by stacking laminar flow vertically. Circulating tumor cells were sorted and captured by the three-dimensional regulation of both magnetic fields in the vertical direction and flow fields in the lateral direction. Using EpCAM-magnetic beads, we achieved sorting and sectional capture of target cells in whole blood and analyzed the surface expression levels of the captured cells, confirming the functionality of the microfluidic chip in sorting and capturing subtypes of circulating tumor cells. This microfluidic chip can also aid in the subsequent subtype analysis of other rare cells.

## 1. Introduction

Circulating tumor cells (CTCs), as a crucial indicator in liquid biopsy, hold significant clinical relevance. With the deepening of the study of CTCs, the results not only show that CTC is an important cause of tumor metastasis [1], but also find that CTCs are heterogeneous [2,3,4,5]; that is, the expression level of tumor cell genes is different, resulting in the existence of tumor cell subtypes. The variability in EpCAM expression is primarily regulated by the process of epithelial–mesenchymal transition (EMT), which is a key mechanism promoting cancer cell invasion, migration, and dissemination [6]. The heterogeneity of CTCs significantly impacts cancer diagnosis, treatment, and monitoring [7,8,9]. It can influence drug resistance, treatment response, cancer progression, and the risk of disease relapse [10]. It is also a major cause of therapeutic failure, as it directly affects therapeutic targets and shapes the tumor microenvironment, leading to the constant reprogramming of the tumor microenvironment [11]. The study on the subtypes of CTCs may bring good therapeutic effect to the treatment of tumor patients.

Currently, there are several methods to isolate CTCs from whole blood: CTC sorting techniques leverage the distinctive properties of CTCs that set them apart from other blood cells. These characteristics primarily encompass both physical and biological properties [12,13]. The physical properties mainly comprise size, density, and deformability [14,15,16], while biological properties mainly consist of specific gene expression and surface proteins [17,18,19,20]. Fluorescence-activated cell sorting (FACS) is another common method for cell sorting but it faces challenges such as reduced cell viability, slow sorting speed, and difficulty in sorting rare cell populations [21].

The emergence of microfluidic chips has recently facilitated the enrichment and fractionation of circulating tumor cells through the integration of microfluidic technology with the special characteristics of these cells [22,23,24]. This includes techniques such as micromembrane-embedded microfluidic chips [16,25,26], dielectrophoretic separation [27,28,29] and immune–magnetic sorting [30,31,32]. While the utilization of microfluidic chips has shown considerable efficiency in the sorting and capture of circulating tumor cells [26,31,33,34,35,36], there is a notable scarcity of research focused on the sorting and capture processes guided by the expression levels of circulating tumor cell markers. This is a significant gap because CTC heterogeneity, manifested through differential expression of surface proteins like EpCAM, plays a crucial role in drug resistance, treatment response, cancer progression, and the risk of disease relapse. The ability to discriminate and study CTC subpopulations based on their marker expression could provide invaluable insights into the complex regulatory mechanisms underlying tumor heterogeneity and disease progression.

Since the level of protein expression on the cell surface is proportional to the number of magnetic bead binding, subtype sorting can be achieved according to the different magnetic forces to which the cells are subjected [37]. Herein, we proposed a microfluidic chip by stacking laminar flow vertically that can not only isolate CTCs from whole blood, but also achieve sectional capture based on variable cell surface marker expression. This approach addresses the existing gap by allowing for the fractionation and characterization of CTC subpopulations, which could provide valuable insights into tumor heterogeneity and pave the way for more personalized and effective cancer therapies. We initially employed immunomagnetic sorting to isolate circulating tumor cells from whole blood, followed by lateral flow-based sectional capture within another layer. In this paper, we validated the microfluidic chip from a flow field perspective and characterized the capture structures within the microchannels. To verify the functionality of the microfluidic chip, we utilized EpCAM as the target surface marker labeled with immunomagnetic beads to sort and successfully achieved sectional capture of target cells from whole blood and quantified the expression of the marker. Furthermore, it was observed that cells captured in different regions exhibited distinct expression levels, aligning with our anticipated outcomes.

## 2. Materials and Methods

### 2.1. Device Design and Fabrication

The entire microfluidic device consists of three layers: the sample layer, the buffer layer, and the top microchannel layer. The sample layer and buffer layer were fabricated by molding processed polymethyl methacrylate (PMMA) templates. The microchannels were fabricated by standard photolithography techniques [38]. The patterns of the chip were designed using AutoCAD 2022 (Autodesk, San Francisco, CA, USA), and SU-8 2050 negative photoresist (Microchem, Newton, MA, USA) was spin-coated onto the pretreated silicon wafer to create a 40 μm high photoresist layer. After spin coating, the baked wafer was exposed to 365 nm UV. The patterned wafer was baked again and developed using a SU-8 developer (Microchem). The polydimethylsiloxane (PDMS, Dow Corning Sylgard 184) was mixed with a curing agent at a 10:1 ratio, poured onto the PMMA templates and patterned wafer, and cured at 80 °C for 2 h. The cured PDMS replicas were peeled off. A puncher with a 1.00 mm inner diameter was used to punch inlet holes for the microchannel of the PDMS device. After plasma treatment, the cured PDMS chips were brought into contact and heated in an oven at 80 °C to achieve an irreversible bonding. For more details see Supplementary Materials (Figure S1).

### 2.2. Cell Culture and Experiment

Two cell lines: MCF-7 (human breast cancer cell lines) and K-562 (human chronic myelogenous leukemia cell lines) were used for our microfluidic chip testing. MCF-7 cells were grown in DMEM containing 10% FBS and 1% penicillin–streptomycin in a humidified atmosphere of 95% CO_2_ at 37 °C. K-562 cells were grown in RPMI-1640 containing 10% FBS and 1% penicillin–streptomycin in a humidified atmosphere of 95%CO_2_ at 37 °C. K-562 cells were stained with Calcein acetoxymethyl ester fluorescent dye (Calcein-AM) (Med Chem Express, Monmouth Junction, NJ, USA) and MCF-7 cells were stained with 1,1′-dioctadecyl-3,3,3′,3′-tetramethylindocarbocyanine perchlorate dye (Dil) (Med Chem Express) for the identification of the different cells in the microchip. All stains were incubated for 15 min at 37 °C. For each cell experiment, 10 μL of K-562 cells at a concentration of 1 × 10^5^ cells/mL (i.e., 10^3^ cells) and 10 μL of MCF-7 cells at a concentration of 1 × 10^5^ cells/mL were mixed with 1 mL of DPBS-EDTA (Dulbecco’s phosphate-buffered saline containing 0.02% Ethylenediaminetetraacetic Acid) to make a sample suspension. To accomplish cell separation and single-cell capture, a system comprising a microscope, a microfluidic chip and two syringe pumps was built (Figure S2). A syringe pump (Fusion 200-X syringe pump, Chemyx Inc., Stafford, TX, USA) was used to withdraw 1 mL of the sample suspension and 1 mL of 0.25% (*w*/*v*) sodium alginate medium (Solarbio, Beijing, China) at a flow rate of 2 mL/h, and injected into the sample layer and buffer layer, respectively. Another syringe pump was used to withdraw 1 mL of 0.25% (*w*/*v*) sodium alginate medium at a flow rate of 3 mL/h and injected into the microchannel layer. All cell lines were obtained from Peking Union Medical College Cell Bank (Beijing, China). 

### 2.3. Cell Labeling with EpCAM Immunomagnetic Beads

CELLection™ Epithelial Enrich Dynabeads (Invitrogen, Waltham, MA, USA, 16,203) were used for specific CTCs’ positive separation. Typically, for every 10^4^ target cells, 5 μL excess dynabeads were added and incubated in the mixer at room temperature for 30 min to ensure that all the binding sites of the target cells were occupied by the magnetic beads. To ensure the accuracy of the experiment, we placed the tubes on a magnetic rack for five minutes, then removed the supernatant. This allowed us to assume that cells not bound to the magnetic beads were removed, leaving only cells bound to the magnetic beads. Furthermore, in the follow-up experiment, 1000 target cells labeled with magnetic beads were spiked into the unprocessed whole blood. 

### 2.4. Preparation of Magnetic Field and Microfluidic Chip

The magnetic field of the entire microfluidic system was generated by combining three NdFeB permanent magnets (10 mm × 10 mm × 20 mm) into a single unit. The magnetic field intensity in the microfluidic system could be controlled by changing the distance between the permanent magnet and the microfluidic chip. Prior to the cell experiment, the microfluidic channels were flushed with ethanol to remove bubbles. A PBS wash was used to remove the residual ethanol. Before performing a spike-in experiment, sodium alginate was used to remove bubbles from the chip and as a buffer in the experiment. 

### 2.5. COMSOL Multiphysics Simulation

Prior to conducting the experiment, we performed simulation validations, including two-dimensional laminar flow simulation and three-dimensional modeling of cell trajectories under the combined effects of magnetic and flow fields. The sample layer and buffer layer constituted a two-dimensional structural model, which utilized the laminar flow module in COMSOL Multiphysics (COMSOL Multiphysics v5.6, COMSOL AB, Stockholm, Sweden). The sample layer and buffer layer each had one inlet and one outlet, with both inlet velocities set equally at 0.001 m/s. To simulate the microenvironment of whole blood, the dynamic viscosity of the fluid was specified to be 0.003 Pa·s and the density of the fluid was specified to be 1060 kg/m^3^. For the simulation of laminar flow, the governing equation is the Navier–Stokes equation. In our simulation, we made the assumptions of fluid incompressibility and constant density and viscosity. With these assumptions, the final form of the Navier–Stokes equation becomes as follows:

(1) $$\begin{array}{c} \frac{\partial v}{\partial t}+\left(v\cdot \nabla \right)v=-\frac{1}{\rho }\nabla p+\mu \nabla^{2}v+f_{v} \end{array}$$

where v is the velocity of the fluid, ρ refers to the density of the fluid, p is fluid pressure, μ is dynamic viscosity, and $f_{v}$ refers to volumetric force of the fluid. The mesh was controlled by the physics fields. We also performed steady-state flow field simulation for the microchannel with a capture structure. A two-dimensional model was established based on the size of the microchannel utilizing a laminar flow model with an inlet velocity set at 0.001 m/s.

In addition, a transient simulation and a steady-state simulation were carried out using COMSOL. A simplified 3D model was established for transient simulation calculations, with the entire geometry including two lateral microchannels (sample layer and buffer layer) and fifteen longitudinal microchannels. Three physical fields, laminar flow, magnetic field without currents, and particle tracing for fluid flow, were used in the simulation. Four types of particles were used to represent high expression, medium expression, and low expression of circulating tumor cells and other blood cells in blood, respectively. And, it was assumed that CTCs were, respectively, conjugated with 100, 10, and 1 magnetic bead. Magnets were placed 8 mm above the chip. The sample layer and buffer layer each had one inlet and one outlet, and negative pressure conditions were set at the outlets of all microchannels. The entire simulation was divided into two studies: first, the magnetic field intensity was calculated; then, the magnetic field intensity data was exported and applied to the fluid flow particle tracking for computational modeling and simulation. In the simulation of particle motion, the cell is subjected to a magnetophoretic force and its equation is

(2) $$\begin{array}{c} F_{m}=2\pi r_{p}^{3}\mu_{0}\mu_{r}k\nabla H^{2} \end{array}$$

and $K=\frac{\mu_{r\cdot p}-\mu_{r}}{\mu_{r\cdot p}+2\mu_{r}}$, where $r_{p}$ is the radius of the particle, $\mu_{0}$ is vacuum permeability, $\mu_{r}$ is the relative permeability of fluid, and $\mu_{r\cdot p}$ is the permeability of the particle. The motion trajectory position data of four types of particles were exported from COMSOL Multiphysics and graphically re-rendered using Python version 3.10.5. The detailed simulation parameters are provided in the Supplementary Materials (Table S1). 

### 2.6. Spiking of Tumor Cells in Whole Blood

Whole blood samples were obtained from healthy donors. A certain number of the MCF-7 cells and K-562 cells were spiked into 1 mL blood based on the experiment design. A syringe pump was used to withdraw the 0.25% sodium alginate medium at a flow rate of 1 mL/h, and it was injected into the sample layer and buffer layer, respectively, to establish a stable laminar flow. After the laminar flow was established, whole blood containing suspended cells was injected into the sample layer. This ensured the formation of a stable laminar flow between the whole blood sample and the sodium alginate medium. During the experiment, the entire buffer was initially injected through the syringe pump to adjust the flow field, allowing the formation of a stable laminar flow between the buffer layer and the sample layer.

### 2.7. Quantitative Analysis of Cell Surface Expression

The relative expression levels of surface marker were measured by counting the number of beads bonding to the cells in the microscope. In order to mitigate variability, we recorded the quantity of cells captured within each individual microchannel, as well as the overall count of cells bound to magnetic beads. Subsequently, we computed the respective averages for a more rigorous analysis.

All photographs including fluorescence images and brightfield images were taken with an EVOS M7000 Imaging System (Thermo Fisher Scientific, Waltham, MA, USA). Scanning electron microscope images were taken on a field emission SEM (GeminiSEM 300, Zeiss, Oberkochen, Germany) at 5 kV.

## 3. Results and Discussion

### 3.1. The Principle of Microfluidic Chips

**Figure 1:**
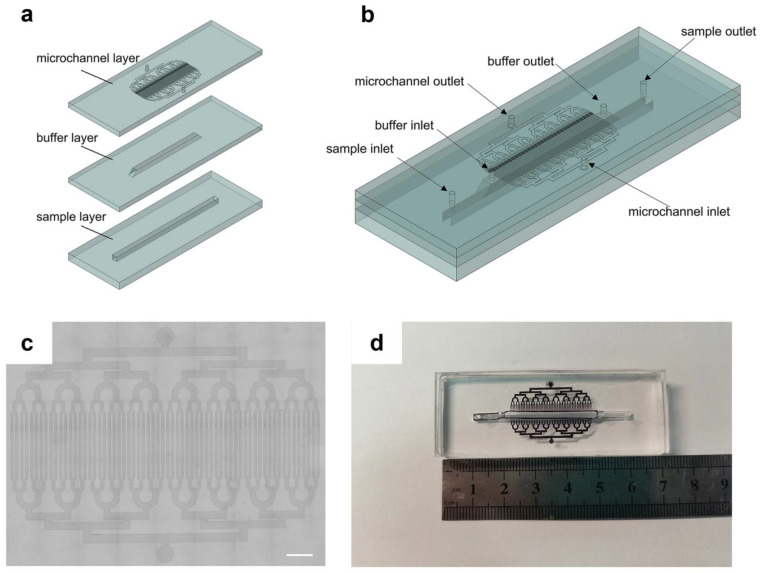
Schematic diagram of the microfluidic chip. (**a**) Three-layer structure of microfluidic chip: sample layer, buffer layer, and microchannel layer. (**b**) The chip including three inlets: sample inlet, buffer inlet, microchannel inlet and three outlets: sample outlet, buffer outlet, microchannel outlet. (**c**) Optical microscopy image of the microchannel layer (scale bar: 2 mm). (**d**) Photograph of the microfluidic chip without placing magnets.

In order to sort and capture subtypes of CTCs, a vertical laminar flow-stacked microfluidic chip was designed for sorting and capturing CTCs. The chip can differentiate between CTC surface antigen expression levels to enable targeted capture. The overall chip structure consists of three layers, as shown in Figure 1a: a bottom sample layer, a middle buffer layer, and a top microfluidic layer. The height and width of both the sample layer and buffer layer are 2 mm. The length of the sample layer is 50 mm, and the length of the buffer layer is 34 mm. The sides of the buffer layer are made into a chamfered form to reduce bubble generation. Each layer has inlet and outlet ports, with the microfluidic layer perpendicular to the lower two layers to create three-dimensional flow channels. The total width of the microchannels is 25.4 mm, and there are 64 microchannels. The width of each microchannel is 250 μm, as shown in Figure 1c. 

**Figure 2:**
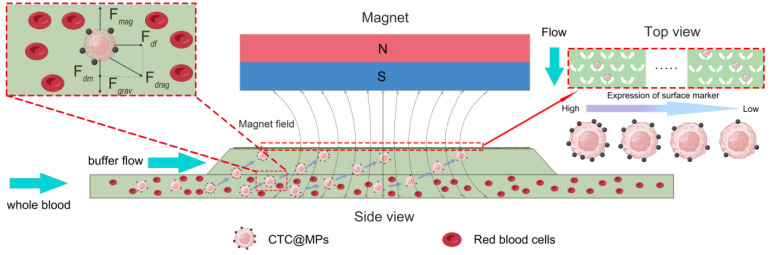
The principle of the microfluidic chip (2D) and the force analysis of cell. The whole process is divided into two stages: vertical separation and longitudinal capture. Cell suspension and buffer flow are introduced from sample inlet and buffer inlet, respectively. CTC@MPs accomplishes vertical separation from sample layer to buffer layer under the magnetic field. The quantity of surface antibodies directly influences the binding of magnetic beads, leading to an increased magnetic force. Consequently, these particles enter distinct positions within the microchannel, effectively achieving the process of capture.

The principle of the microfluidic chip is shown in Figure 2. The chip operates through a two-stage process: (i) isolation of CTCs from the sample layer using immunomagnetic separation, and (ii) capture of CTCs within the microchannels under flow conditions. The first stage involves the vertical separation of CTCs from the sample layer utilizing a magnetic field above the chip to segregate and aggregate the magnetic particles-bound CTCs (CTCs@MPs). The buffer layer and sample layer establish stable laminar flow to prevent non-target blood cells, including red and white blood cells, from entering the microfluidic layer under the flow field. These non-target cells and uncaptured components are discarded. In the second stage, CTCs exhibit differential surface marker expression, binding varying numbers of magnetic beads. Higher surface expression leads to increased bead binding and greater magnetic force. After the CTCs enter the microchannel vertically, the lateral flow field can drive the cells to continue to move. From a top view of the chip, cells with high marker expression are captured in microchannels proximal to the sample inlet, while cells with low marker expression reach the distal microfluidic channels due to weaker magnetic forces. Consequently, the expression of cells decreases along the inlet-to-outlet axis. Within the microchannels, the U-shaped capture structure is much smaller than the cell dimensions, trapping target cells.

### 3.2. Force Analysis of Cells

Under an active external field, the motion of a magnetic bead-bound cell is dominantly regulated by three forces: the drag force from the flow field ($F_{drag}$), the net gravitational force ($F_{grav}$) considering buoyancy, and the force by the external magnetic field ($F_{mag}$).

The drag force can be decomposed into two components in the horizontal and vertical directions, denoted as horizontal drag force $\left(F_{df}\right)$ and vertical drag force ($F_{dm}$), respectively. The drag force is determined by the fluid viscosity, particle size, and relative velocity, which can be evaluated by the Stokes equation [39]:

(3) $$\begin{array}{c} F_{drag}=6\pi \eta R_{eq}v\; \end{array}$$

where η is the viscosity of fluid, $R_{eq}$ is the equivalent radius of the target cells bonding with magnetic beads, and v is the velocity of the cell relative to the fluid.

The net gravitational force can be expressed by the following equation:

(4) $$\begin{array}{c} F_{grav}=V_{p}\left(\rho_{p}-\rho \right)g \end{array}$$

where $V_{p}$ represents the volume of the particle, $\rho_{p}$ is the density of the particle, ρ refers to the density of the fluid, and g is the gravitational acceleration.

Since the magnetic beads are specifically bound to the cells in the flow field, the magnetic moment of individual dispersed beads is 0. However, under a non-uniform external magnetic field, the beads will form a magnetic moment and be subjected to the magnetic force, which can be expressed as follows [40]:

(5) $$F_{mag}=N\left(\chi_{p}-\chi_{m}\right)\frac{V_{p}}{\mu_{0}}\left(B\cdot \nabla )B\right.=N\frac{V_{p}\Delta \chi }{2\mu_{0}}\nabla B^{2}\;\Delta \chi =\chi_{p}-\chi_{m}$$

where N is the number of magnetic beads bound to the cell, $\chi_{p}$ is the magnetic susceptibility of the beads, $\chi_{m}$ is the magnetic susceptibility of the fluid medium, $\mu_{0}$ is the vacuum permeability, and B is the magnetic flux density of the applied magnetic field. Since $\chi_{p}$ >> $\chi_{m}$, the magnetic force can be expressed as follows:

(6) $$F_{mag}=N\frac{V_{p}\chi_{p}}{2\mu_{0}}\nabla B^{2}$$

Due to the relatively large magnetic field generated by the permanent magnet utilized in this microfluidic sorting and capture system (~600 mT), the forces acting on target cells can be simplified to include only drag force and magnetic force, with net gravitational force being negligible [41]. Therefore, when cells are in a state of force equilibrium, they experience the effects of magnetic field force and drag force exclusively. At this juncture, the velocity of the cells in the vertical direction (u) can be expressed as follows:

(7) $$F_{mag}=N\frac{V_{p}\chi_{p}}{2\mu_{0}}\nabla B^{2}$$

Assuming the initial position of the cells in the y-axis direction is $x_{0}$, and a total height H encompassing both the buffer and sample layers, the time t required for cells to reach the microchannel layer can be denoted as follows:

(8) $$t=\int_{x_{0}}^{H}\frac{dx}{u}$$

Derived from Equations (7) and (8), it can be observed that cells with higher expression levels exhibit a faster entry into the upper microchannel, resulting in shorter transit times. Consequently, these cells generate shorter displacements in the horizontal direction, facilitating their capture closer to the entrance. As the cellular expression levels decrease sequentially, the captured positions gradually shift away from the entrance.

### 3.3. Cell Movement Trajectories in COMSOL Simulation

**Figure 3:**
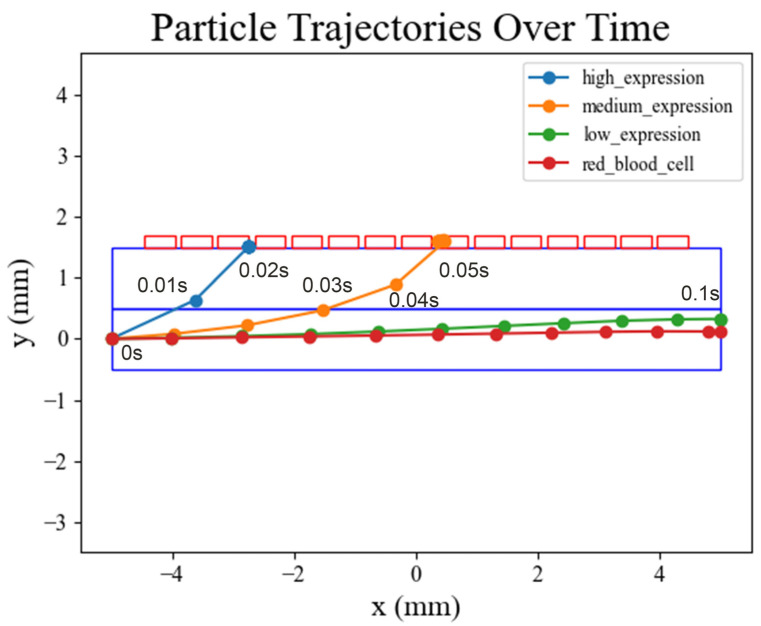
Simulation of cell trajectories over time on COMSOL. Cells with high levels of expression entered the upper microchannel at 0.02 s, while those with medium levels of expression entered at 0.05 s. In contrast, cells with low levels of expression and red blood cells were eventually washed out of the sample stream at 0.1 s.

After completing the theoretical proof, we utilized COMSOL for simulation validation. Four types of particles with different properties were used to simulate cell motion. As is shown in Figure 3, the results show that circulating tumor cells with the highest expression (i.e., those with the largest number of bound magnetic beads) experience the greatest magnetic force and are the first (at 0.02 s) to penetrate the buffer layer and reach the front end of interface between the buffer layer and the microchannel. And then, they enter the microchannel preferentially under the effect of the microchannel flow. Circulating tumor cells with medium expression have a smaller force relative to the highly expressed cells and rise more slowly. Under the effect of the lateral flow, they eventually reach the middle position of the interface between the buffer layer and the microchannel and enter the microchannel under the effect of the longitudinal flow. Circulating tumor cells with low expression, having bound the fewest magnetic beads, experience insufficient magnetic force to overcome the buffer layer hydrodynamics. As a result, they cannot be separated and exit through the sample layer outlet. Other blood cells and circulating tumor cells with low biomarker expression experiencing no magnetic force directly exit through the sample layer outlet.

### 3.4. Characterization of the Microfluidic Chip

**Figure 4:**
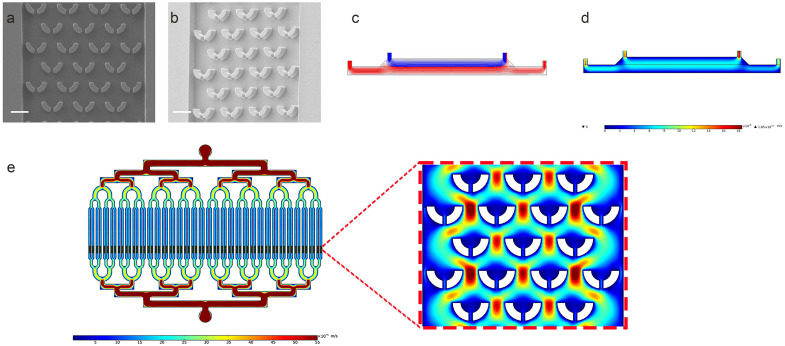
Characterization of the microfluidic chip. (**a**,**b**) Partial view of the SEM image of capture structure. (**a**) U-shaped capture structure in two dimensions. (**b**) U-shaped capture structure in three dimensions (scale bar 20μm). (**c**) Streamline between the buffer layer and the sample layer. (**d**) Velocity field between the buffer layer and the sample layer. (**e**) Simulation diagram of overall and locally magnified flow field of microchannel.

We conducted characterization primarily in the following aspects: (i) Capture structures in the microchannels. (ii) Flow field analysis between the buffer layer and sample layer, as well as within the microchannels. As shown in Figure 4, U-shaped capture structures were imaged under an electron microscope, providing perspectives from both two-dimensional and three-dimensional angles. U-shaped capture structures were positioned proximal to the outlet of each microchannel for cell capture. The U-shaped capture structure was designed with an inlet opening for initial cell capture, as well as a narrower outlet opening of 5 μm for cell capture with fluidic flow. In addition, simulations of the flow between the buffer layer and sample layer were conducted using COMSOL to model the motion of cells under the combined effects of magnetic and flow fields. In the two-dimensional simulation, a stable laminar flow could be established between the sample and buffer layers, ensuring that non-target cells in the sample layer would not enter the microchannel due to the flow field. The streamlines between the flow fields are shown in Figure 4d, with the two phases forming laminar flows that do not interfere with each other. We utilized PDMS to fabricate a dual-layer chip comprising only the buffer layer and the sample layer. The infusion of fluorescein sodium was employed to validate the stability between the two flow fields. For details, refer to Supplementary Materials (Figure S3). After performing flow field simulations for the microchannels, as illustrated in Figure 4e, comprehensive as well as localized velocity field images encompassing the U-shaped structure were captured. Furthermore, we validated the capturing capability of the microfluidic layer. We directly introduced cells without bound magnetic beads into a single-layer microchannel to observe the cell capture process by the U-shaped structure. For details, please see Supplementary Materials (Figure S4).

### 3.5. Cell Sorting and Capture Using Microfluidic Chip

**Figure 5:**
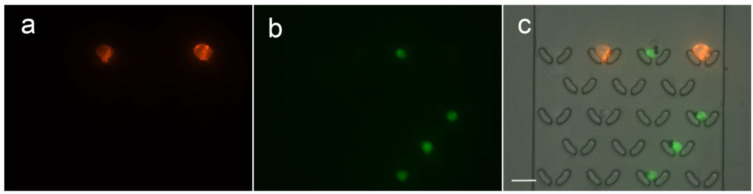
Fluorescent microscopy of captured cells in the microchannel. (**a**) MCF-7 stained with Dil (Red), (**b**) K-562 stained with Calcein-AM (Green), and (**c**) merged image of (**a**,**b**) and bright field. (Scale bar 30 μm).

Elevated EpCAM levels in cancers make it a crucial marker for detecting circulating tumor cells, influencing cancer diagnosis and prognosis [42]. Additionally, EpCAM’s involvement in cellular functions and its role as a biomarker contribute significantly to cancer research and diagnostics, guiding prognosis assessment and targeted therapy selection [43,44,45]. Consequently, we have chosen EpCAM as the sorting marker for subsequent cell sorting and capture experiments. In the experiment, the immunomagnetic beads we utilized offer the advantage of having a dedicated bead elution solution. Following the experiment, captured cells can be collected and subjected to elution with the elution solution for subsequent culture. A mixed suspension of K-562 cells and MCF-7 cells combined with immunomagnetic beads was prepared for cell sorting and capture. Due to the different surface expression levels and cell types of K-562 and MCF-7 cells, this demonstrates the universality of the sorting and capturing functions of the microfluidic chip. Using the microfluidic chip, we successfully captured individual K-562 and MCF-7 cells, with K-562 stained in green fluorescence using Calcein-AM and MCF-7 stained in red fluorescence using Dil. Figure 5 shows the results of fluorescent images of the captured cells. Through the images, the visual appearance of the binding between cells and magnetic beads, as well as the morphology of cells captured by the U-shaped structure, can be clearly observed. The achievement of effective single-cell capture is evident in the cellular experiments. Despite the differences in expression levels between K-562 and MCF-7 cells, it is apparent that they can still undergo concurrent sorting and capture, demonstrating the system’s capability for the successful isolation of individual cells. However, due to variations in the digestion process of adherent cells by different operators, discrepancies in cell digestion may occur. Adherent cells may exhibit poor dispersion, resulting in difficulties in single cell capture. Therefore, ensuring the dispersion of adherent cells is essential during the experiment.

### 3.6. Quantification of Surface Expression Levels on Captured Cells

**Figure 6:**
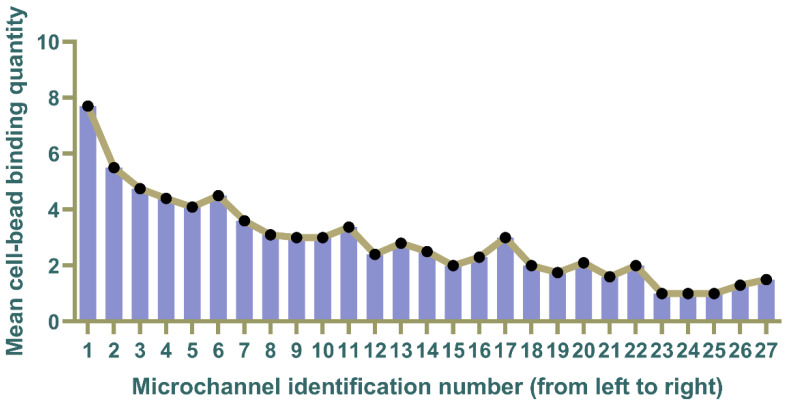
The average number of cells bound to magnetic beads at various microchannels (from left to right) Channel 1 is closest to the inlet, and as the number increases, the microchannels progressively move away from the inlet, with Channel 27 being the farthest from the inlet. The highest average quantity of cells bound to magnetic beads is 7.7, while the lowest is 1.

After achieving the sorting and capture of cell mixtures, we attempted to isolate the captured target cells from whole blood. 1 mL of untreated whole blood containing cell suspensions of K-562 and MCF-7 cells was introduced into the microfluidic chip, concomitant with the utilization of sodium alginate as a buffering solution. For spike-in experiments, the figures can be found in Supplementary Materials (Figure S5). When performing CTC sorting and capture with red and white blood cells as background cells in a microfluidic chip, the importance of establishing laminar flow between the buffer layer and the sample layer becomes evident. The buffer layer effectively hinders the entry of red and white blood cells into the upper microchannel, preventing their capture due to the influence of the flow field. After the completion of the sorting and capture process, we quantified the expression of cell surface EpCAM by statistically counting the number of cells bound to magnetic beads in each microchannel in the entire microfluidic layer. The microfluidic layer comprises a total of 64 microchannels, and within these, we observed cell capture in 27 microchannels. Subsequently, each of these microchannels was sequentially numbered from 1 to 27. Cells at different locations were bound by varying quantities of magnetic beads. We selectively captured images of cells at three distinct positions, representing high expression, moderate expression, and low expression, respectively. Detailed images can be found in Supplementary Materials (Figure S6). We systematically recorded the total number of cells bound to magnetic beads in each microchannel and calculated the average. The results of the average binding quantity of magnetic beads are depicted in Figure 6. Microchannels with smaller numbers, which are closer to the inlet, exhibit heightened surface expression of cells, resulting in the highest average number of bound magnetic beads. Conversely, as the channel numbers increase further from the inlet, the average bead quantity gradually decreases. Although some microchannels show deviations from a strict decrease in bead quantity with increasing channel numbers, the overall trend demonstrates a decline. The maximum average bead binding quantity for cells is recorded at 7.7, while the minimum is 1, emphasizing the indispensable role of at least one bead for effective sorting and capture. In addition, to evaluate the performance of our CTC isolation method, we conducted spiking experiments using whole blood samples spiked with MCF-7 cells. In each experiment, 100 MCF-7 cells were introduced into the blood sample. Detailed information on separation efficiency and purity can be found in the supplement information (Figure S7). While simulations indicate that cells bound to a solitary magnetic bead cannot be effectively isolated and captured under the corresponding magnetic field, practical experimentation reveals that through the manipulation of the distance between the magnet and the microfluidic chip, even cells with a single magnetic bead attachment can be successfully separated and captured within the magnetic field. Furthermore, in simulation scenarios, discernible differentiation effects manifest predominantly when there is an order of magnitude alteration in the quantity of magnetic beads. In actual experimental settings, however, the lack of a substantial disparity in the quantity of bound magnetic beads results in less conspicuous distinctions in expression levels. And since the external magnetic field gradients are not entirely identical, even when the same number of magnetic beads are combined, there are variations in the magnetic forces exerted on the cells, leading to differences in the final capture positions. Nevertheless, the overall expression level of the cells is still on a declining trend. This confirms the feasibility of the microfluidic chip.

## 4. Conclusions

In this work, a simple but effective microfluidic chip was developed for the sorting and capture of subtypes of circulating tumor cells. Different from traditional microfluidic cell magnetic sorting chips, our method only requires collaborative interaction between the laminar flow field and magnetic field to achieve straightforward subtype sorting and capture of circulating tumor cells. We demonstrated the feasibility of subtype sorting and capturing of circulating tumor cells using a microfluidic chip through analysis of cellular force and COMSOL simulation. Employing EpCAM immunomagnetic beads, we successfully sorted and captured two types of cells, K-562 and MCF-7 cells, with different surface expressions. In the context of whole blood, subtype sorting and capturing of CTCs are also feasible. The surface expression level analysis on the captured cells demonstrated the capability of subtype sorting and single-cell capture. These results confirm the functionality of the microfluidic chip in sorting and capturing subtypes of circulating tumor cells. This lays the foundation for subsequent subtype analysis of circulating tumor cells. Moreover, this microfluidic chip can also subtype sort and capture other rare cells in the blood and genome-edited cells. This microfluidic chip has a great potential for application in the field of clinical diagnosis and treatment.

In summary, the microfluidic sorting and capture platform demonstrates commendable performance and broad application prospects. This platform efficiently sorts and captures rare cells, which is of significant clinical value for early tumor diagnosis and liquid biopsy. More importantly, the platform can capture cells with low expression of surface marker, addressing the limitations of existing sorting technologies and contributing to the exploration of the role of cellular heterogeneity in tumor metastasis. Additionally, based on the recognition of cell surface markers, the platform achieves precise sorting of different cell subpopulations, providing a powerful tool for the systematic study of cell function.

Furthermore, the platform provides a good method for explaining the expression regulation mechanism of EpCAM. EpCAM plays a crucial role in the development of tumors, but its expression shows significant heterogeneity, and the molecular regulatory mechanisms need to be elucidated. Efficient sorting and capturing of cells with different levels of EpCAM expression will help reveal the molecular basis of EpCAM heterogeneity expression, providing new biomarkers for early tumor detection and personalized therapy. Additionally, a deeper understanding of EpCAM regulation mechanisms will also provide new insights and strategies for the development of drugs targeting EpCAM, promoting the development of precision therapy for tumors. In the future, this platform holds the potential to conduct therapeutic trials for studying the expression regulation mechanism of EpCAM and enabling new avenues for precision therapies.

## Data Availability

Data are contained within the article or Supplementary Materials.

## Supplementary Materials

The following supporting information can be downloaded at: https://www.mdpi.com/article/10.3390/mi15040542/s1, Figure S1: The fabrication of the microfluidic chip; Figure S2: Photograph of cell sorting and capture system; Table S1: COMSOL simulation parameters; Figure S3: The laminar flow established between the buffer layer and the sample layer; Figure S4: The process of cells being captured by the U-shaped structure; Figure S5: Whole blood testing; Figure S6: The capture of cell with different expression levels. Figure S7: Sorting efficiency and purity of CTC isolation.
