# Supplementary material for: Precision Phenotypic Profiling and Capture of Circulating Tumor Cells via a Vertical Laminar Flow-Stacked Microfluidic Chip
Source: Micromachines (Basel). 2024 Apr 18;15(4):542. doi: 10.3390/mi15040542 (PMC11051858; doi:10.3390/mi15040542)
Supplement: Supplementary file 1 [file micromachines-15-00542-s001.zip › micromachines-2938185-supplementary.pdf]

Precision Phenotypic Profiling and Capture of Circulating Tumor Cells via a Vertically Laminar Flow Stacked Microfluidic Chip

Xinping Zhang [1], Yuan Ma [2,*], Yujiao Wang [2], Zhenwei Liang [2], Xuanhe Zhang [2], Yiqing Chen [2], Qingyi Wang [1], Hua Qin [1,*] and Jiadao Wang [2,*]

1 School of Mechanical-electronic and Vehicle Engineering, Beijing University of Civil Engineering and Architecture, Beijing 102616, P.R. China

2 Department of Mechanical Engineering, Tsinghua University, Beijing 100084, P.R. China

*Corresponding author E-mail: jdwang@mail.tsinghua.edu.cn, qinhua@bucea.edu.cn, yuanma@tsinghua.edu.cn

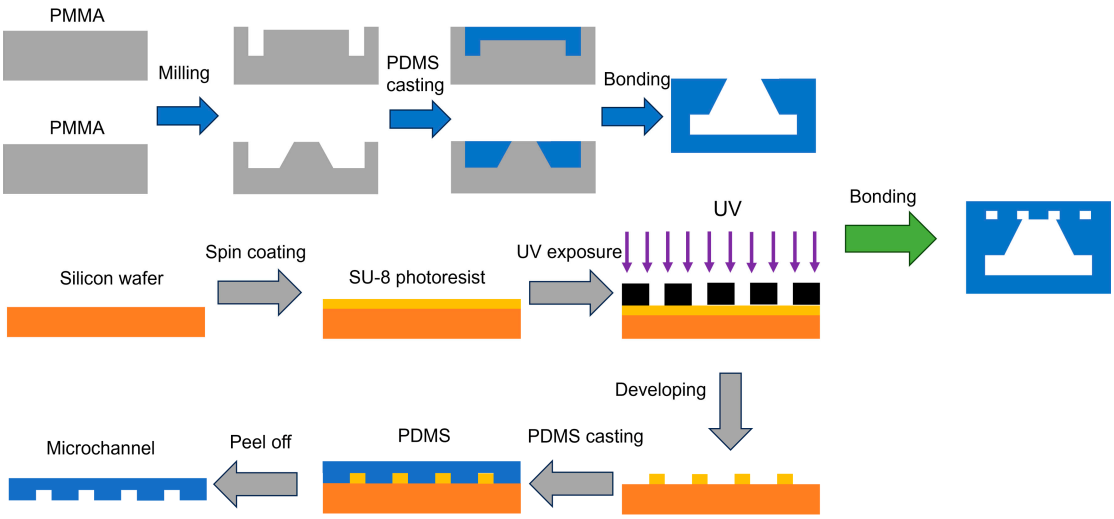

Figure S1 The fabrication of the microfluidic chip. By milling the PMMA board, a master mold was created. Subsequently, PDMS was poured into the master mold to create the sample layer and buffer layer of the microfluidic chip. The microchannel portion was produced through traditional photolithography, using a spin coater to apply SU-8 2050 photoresist at a speed of 3000 rpm on a silicon wafer, resulting in a photoresist height of 40 µm. The wafer was then pre-baked at 65°C and 95°C for 5 minutes and 15 minutes, respectively. Following this, the wafer underwent photolithography with a mask at an exposure power of 40 W/cm² for 4 seconds,

followed by post exposure bake at 65°C for 1 minute and 95°C for 5 minutes. Finally, the developed photoresist created a template for the microchannels. PDMS was poured onto the template to form the microchannel layer. The microchannel layer was then bonded with the buffer layer and the sample layer to obtain the final microfluidic chip using plasma treatment for 20s at a power of 50W.

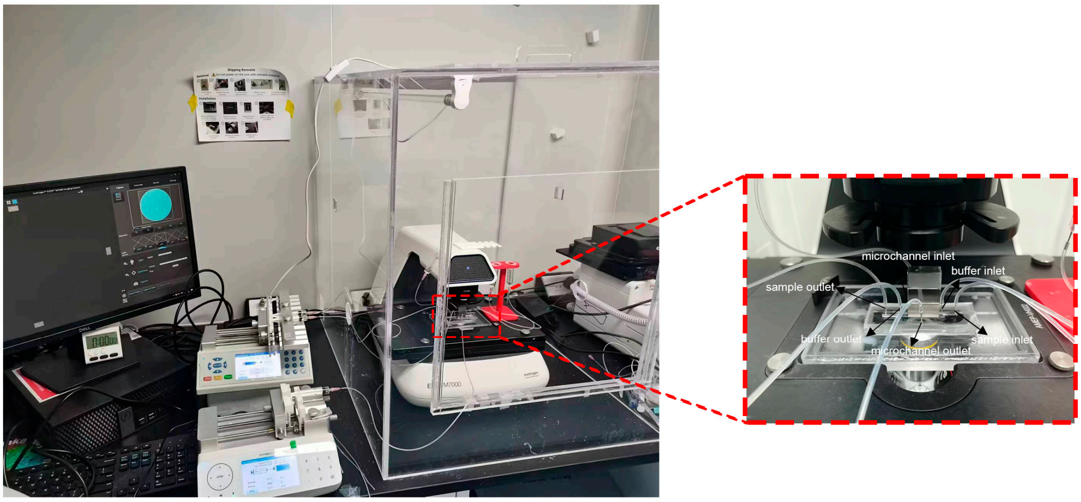

Figure S2 Photograph of cell sorting and capture system based on cell surface marker expression levels using a three-dimensional microfluidic chip.

Parameters	Value	Description
mu	0.004 Pa·s	Fluid viscosity
g	9.8 m/s^2	Gravity acceleration
N_1	100	The number of magnetic beads bound to cells with high expression
N_2	10	The number of magnetic beads bound to cells with medium expression
N_3	1	The number of magnetic beads bound to cells with low expression
D	10μm	Size of cell
d	2.8μm	Size of magnetic bead
χ_p	1.4e-3	Magnetic susceptibility of bead
mu0	1.2566e-6 H/m	Vacuum permeability

Table S1 COMSOL simulation parameters

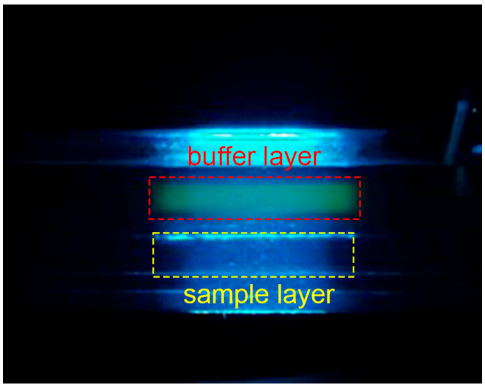

Figure S3 The laminar flow established between the buffer layer and the sample layer, wherein the sample layer is infused with fluorescein sodium, emitting green fluorescence under the green fluorescence channel.

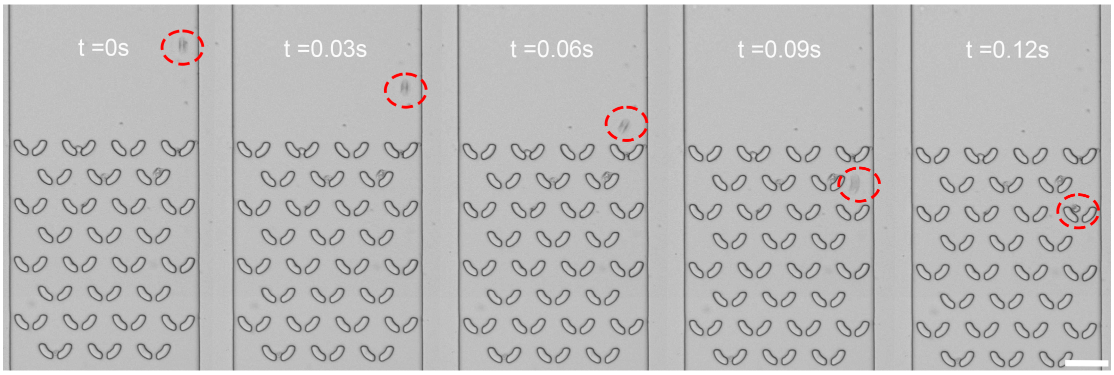

Figure S4 The process of cells being captured by the U-shaped structure. Commencing the time count from the point when cells entered the lens, marked as 0 seconds, the recording initiated with captures captured at intervals of 0.03 seconds. Notably, at 0.12 seconds, successful capture of the cell by the U-shaped structure was achieved (scale bar: 50μm).

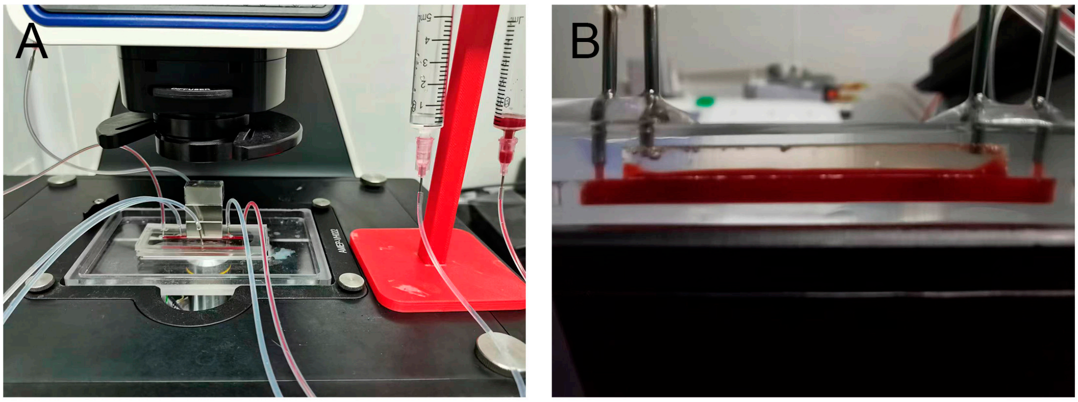

Figure S5 (A) Experimental depiction of whole blood testing and (B) the laminar flow phenomenon generated by the interplay of blood and sodium alginate buffer solution in the dual-layer chip.

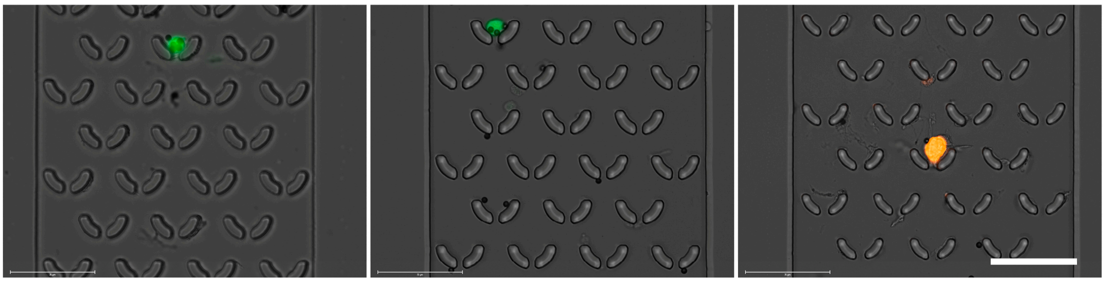

Figure S6 Cells with different expression levels are captured at different positions in the channel, where (A) cell with high expression bound to 5 magnetic beads, (B) cell with medium expression bound to 3 magnetic beads, and (C) cell with low expression bound to 1 magnetic bead. (Scale bar: 75μm)

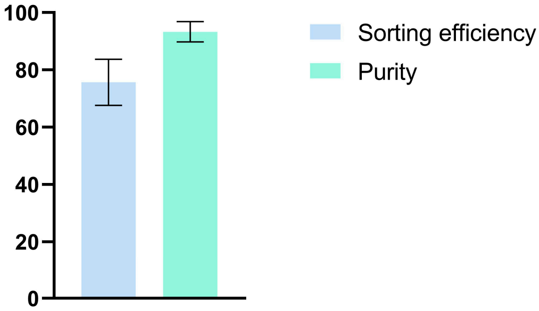

Figure S7 The sorting efficiency and purity of CTC isolation. The sorting efficiency is about 75.6%

and the purity is about 93.3%.
